# Blood Pressure Increase in Hypertensive Individuals During Resistance Training Protocols With Equated Work to Rest Ratio

**DOI:** 10.3389/fphys.2020.00481

**Published:** 2020-06-29

**Authors:** Anderson Caetano Paulo, Claudia L. M. Forjaz, Décio Mion, Giovanio V. Silva, Silvana Barros, Valmor Tricoli

**Affiliations:** ^1^Academic Department of Physical Education, Federal Technological University of Paranná, Curitiba, Brazil; ^2^School of Physical Education and Sport, University of São Paulo, São Paulo, Brazil; ^3^General Hospital, University of São Paulo, São Paulo, Brazil

**Keywords:** weight lifting, cardiovascular response, rest interval, perceived exertion, finger photoplethysmography

## Abstract

**Introduction:** Despite growing evidence regarding the benefits of resistance training in hypertension, the large and abrupt rise of systolic blood pressure (SBP) observed during resistance exercise execution has resulted in concern about its safety. However, the manipulation of the resistance training protocol (RTP) organization, maintaining the work to rest ratio equated between protocols (W:R-equated), may reduce the SBP increase.

**Purpose:** To compare cardiovascular responses during two W:R-equated RTPs (3 × 15:88 s vs. 9 × 5:22 s – sets × reps: rest between sets) performed in exercises for the lower and upper limbs.

**Methods:** Twelve medicated hypertensives (48 ± 8 years) randomly performed two RTPs in the bilateral leg extension (BLE) and unilateral elbow flexion (UEF) exercises at 50% 1RM. Increases (Δ) of SBP, heart rate (HR) and rate pressure product (RPP) during the exercises were measured by photoplethysmography.

**Results:** In both BLE and UEF exercises, Δ SBP was significantly greater during 3 × 15:88 s than 9 × 5:22 s (peak values: BLE = + 84 ± 39 vs. + 67 ± 20 mm Hg, and UEF = + 46 ± 25 vs. + 37 ± 18 mm Hg, respectively, both *p* < 0.05). ΔHR and ΔRPP were significantly higher in the 3 × 15:88 s than 9 × 5:22 s in BLE (peak values + 45 ± 17 vs. + 30 ± 8 bpm, and + 15,559 ± 5570 vs. + 10,483 ± 2614 mm Hg. bpm).

**Conclusion:** In medicated hypertensives, a RTP combining more sets with less repetitions per set and shorter rest intervals between sets (i.e., 9 × 5:22 s) produced a smaller increase in cardiovascular load (ΔSBP, ΔHR and ΔRPP) during its execution than a protocol with fewer longer sets (i.e., 3 × 15:88 s).

## Introduction

Systemic arterial hypertension is characterized by sustained elevation of systolic (SBP > 140 mm Hg) and/or diastolic (DBP > 90 mm Hg) blood pressures (Lenfant et al., 2003; Mancia et al., 2013; Malachias et al., 2016). It affects about 37% of adults worldwide, and is one of the major modifiable risk factors for cardiovascular disease, morbidity, and mortality (Martínez-Rueda et al., 2019). Resistance training is recommended as a complementary exercise therapy for hypertension treatment (Chobanian et al., 2003; Lenfant et al., 2003; Volaklis and Tokmakidis, 2005; Williams et al., 2007; Cornelissen et al., 2011; Mancia et al., 2013; Thompson et al., 2013; Malachias et al., 2016; Martínez-Rueda et al., 2019). Although the effects of this type of training on hypertensives’ BP is controversial (Cornelissen et al., 2011), resistance training increases muscle strength (Williams et al., 2007), which is strongly associated with reduced mortality (Williams et al., 2007), and ameliorates comorbidities usually associated with hypertension (Colberg et al., 2010).

Recommendations of resistance training protocols (RTP) for hypertensives include moderate intensity (40–60% 1RM), high number of repetitions per set (10–15 repetitions), long rest intervals between sets (>60 s), and one to three sets per exercise (Williams et al., 2007; Malachias et al., 2016). In general, these recommendations aim to attenuate the acute BP increase observed during the resistance exercise execution that is greater in hypertensives than normotensives (Nery et al., 2010). This is an important concern because hypertensives have a higher risk of developing aneurysms, and an abrupt and exacerbated increase of BP, as observed during RTP execution, may lead to their rupture (Vlak et al., 2012). In fact, although the risk of an undesired cardiovascular event during resistance exercise is low (Williams et al., 2007), cases of subarachnoid hemorrhage (Haykowsky et al., 1996) and aortic dissection (Hatzaras et al., 2007) have been reported in the literature.

It is known that BP increase during exercise execution can be changed by manipulating RTP variables (Mitchell et al., 1980; MacDougall et al., 1985; Lamotte et al., 2005a, b, 2010). In a previous study (Paulo et al., 2019), we compared three RTPs composed of 45 repetitions and 176 s of rest with equated work to rest ratio (W:R-equated): (i) 3 × 15:88 s (sets × repetitions: rest between sets), (ii) 9 × 5:22 s, and (iii) 45 × 1:4 s. Interestingly, the RTP that had characteristics similar to the hypertension guidelines’ recommendations (3 × 15:88 s) promoted the highest BP peaks during its execution, while the RTP with intermediate sets and intervals (9 × 5:22 s) caused the lowest peaks with responses associated with rating of perceived exertion (RPE) and blood lactate accumulation. These findings suggest that the intermediate protocol may be more appropriate for hypertensives than the usual one. Actually, other studies have also compared BP increase during W:R-equated RTPs (Río-Rodríguez et al., 2016; Mayo et al., 2017; Paulo et al., 2019), but none of them was conducted with hypertensive individuals.

Hypertension causes multiple structural and functional cardiovascular changes that modify responses to exercise, producing not only a greater increase of BP during resistance exercise execution, but also a slower recovery during the intervals (Nery et al., 2010); which may distinctly change BP responses to different RTPs. In addition, the use of anti-hypertensive medications also affects BP response to RTPs (Gomides et al., 2010a; Souza et al., 2015; Ash et al., 2017). Thus, the hypothesis that a W:R-equated RTP with an intermediate number of sets and rest intervals may produce a lower BP increase during its execution than the RTP recommended in the hypertensives’ guidelines, should be tested.

In addition, for health improvement, resistance training may include exercises for the whole body, including upper and lower limbs (Williams et al., 2007). However, it is rare to find a study that has evaluated BP response during RTP for the upper limbs, but Lewis et al. (1985) have reported a smaller BP increase in comparison with the lower limbs exercises; which has been attributed to the smaller muscle mass involved. Nevertheless, the study by Lewis et al. (1985) was also conducted with normotensives and responses may be different in hypertensives.

Therefore, the aim of this study was to compare, in medicated hypertensives, the cardiovascular responses during the execution of two W:R-equated RTPs (3 × 15:88 s and 9 × 5:22 s) performed with lower and upper limb exercises.

## Materials and Methods

### Participants

Essential hypertensives were invited for the study. Those who wanted to participate were informed of the procedures and risks, and signed an informed written consent approved by the Institutional Research Ethics Committee (number 110554). Participation criteria included; being aged between 30 and 60 years; taking up to three antihypertensive medications; having a SBP/DBP lower than 160/105 mm Hg; having a body mass index (BMI) lower than 35 kg. m^–2^; have no comorbidities (other diseases) and performing less than 150 min of physical activity per week. To assure the fulfilling of these criteria, all volunteers were examined in the Hypertension Unit of the General Hospital in the Faculty of Medicine of the University of São Paulo.

### Familiarization and Experimental Procedures

All participants who fulfilled the study criteria underwent familiarization sessions to the 1 repetition maximum (1RM) assessment procedures and to the study protocol in addition to two experimental sessions. They were instructed to keep the same routine in the preceding day of all sessions. Sessions were conducted between 6 and 10 a.m., in a temperature controlled laboratory (20–22°C), and with an interval of at least 72 h between them. Additionally, the participants were instructed to take their medications before the sessions as prescribed by their own physicians. The recommendations were checked before the beginning of the experimental sessions.

In each session, two exercises were performed: bilateral leg extension (BLE) on a leg extension machine (Physicus, model PHA-23, Auriflama, SP, Brazil) and unilateral elbow flexion (UEF) with the dominant limb on a Scott bench (Gantry, model FW3050, Itatiba, SP, Brazil). The order of the exercises’ execution was randomized in the first session and was kept constant in the following sessions for the same volunteer. In addition, the equipment was adjusted to the participant’s body size and adjustments were replicated in all subsequent sessions.

In the first session for 1RM assessment, participants performed two BLE or two UEF warm-up sets. During the first set, volunteers performed five repetitions with 50% of the estimated 1RM. In the second set, they performed three repetitions with 70% of the estimated 1RM, with 3 min intervals between sets. After the second warm-up set, volunteers rested for 3 min. Then, they had up to five attempts to reach their 1RM. An interval of 3 min was allowed between the maximal attempts. After finding the value of 1RM for the first exercise, an interval of 3 min was given and the same procedures were performed for the other exercise. In the second session, the 1RM tests were repeated, and if a difference greater than 5% of the previous 1RM value was detected, a new test session was scheduled. This procedure was repeated until 1RM load was stabilized with a difference of less than 5% between tests.

Afterward, the participants underwent a familiarization session to the RTPs and the other experimental procedures. During this session, they executed the BLE and the UEF at 50% of 1RM with the rhythm controlled by a metronome (2 s: 2 s). Additionally, as the participants had no previous experience with RPE scale, during this familiarization session, they learned and experienced how to anchor and classify RPE for BLE and UEF using the chosen scale.

**FIGURE 1:**
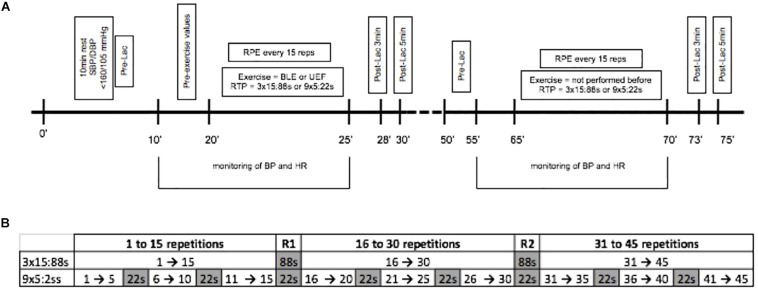
Experimental procedures and description of the two resistance training protocols. **(A)** Lac, lactate; RPE, rate of perceived exertion; BLE, bilateral leg extension exercise; UEF, unilateral elbow flexion exercise; RTP, resistance training protocols; 3 × 15:88 s and 9 × 5:22 s (sets × reps: rest between sets); BP, blood pressure, HR, heart rate. **(B)** In each protocol, repetitions are shown in white and rest intervals in gray. R1, First common rest intervals after the 15th repetition. R2, Second common rest interval after the 30th repetition.

Finally, the participants underwent two experimental sessions (3 × 15:88 s and 9 × 5:22 s) conducted with a crossover randomized design. The experimental procedures of each session are shown in Figure 1. In the first experimental session, after arriving at the laboratory, the participants sat on the equipment for the first exercise (BLE or UEF). Then, they rested for 10 min (pre-exercise period), performed the selected RTP (3 × 15:88 s or 9 × 5:22 s) and rested for another 5 min (post-exercise period). Afterward, a 20 min interval was allowed for the complete return of BP and HR to the pre-exercise values; and then participants repeated all procedures with the second exercise. In the second experimental session, all procedures were repeated using the other RTP (3 × 15:88 s or 9 × 5:22 s).

Both RTPs were designed to have equated work to rest ratio, i.e., 45 repetitions and 176 s of passive rest. In the 3 × 15:88 s, participants executed three sets of 15 repetitions with 88 s of interval between sets; while in the 9 × 5:22 s, they executed nine sets of five repetitions with 22 s of interval between sets (Figure 1). RTPs were executed with a workload of 50% 1RM and a concentric to eccentric rhythm of 2 s: 2 s. For comparison between the protocols, data were analyzed every three blocks of 15 repetitions (i.e., 1–15 rep, 16–30 rep, and 31–45 rep) and in the two common rest intervals that occurred between the 15th and 16th (R1) and 30th and 31th (R2) repetitions.

During the experimental sessions, beat-to-beat BP was measured through photoplethysmography (Finometer^®^, Finapres Medical Systems, Amsterdam, Netherlands) and heart rate (HR) through electrocardiography (ADinstruments, MLA0115/S ECG 12, New Zealand). Both signs were synchronized with a data acquisition system (LabChart Pro version 7.2.5, CA, United States) using a sampling rate of 500 Hz/channel. Rate pressure product (RPP) was calculated as the product of SBP and HR. Pre-exercise values were calculated by the average of 10 min. Responses during the RTPs were calculated by the area under the curve and by the greatest changes. The area under the curve was calculated considering all measurements done during the whole execution of each RTP (GraphPad Prism 7). The greatest changes (Δ) were calculated by the difference between the highest values obtained every 15 repetitions block (i.e., 1–15 rep: 16–30 rep, and 31–45 rep) and the pre-exercise value. In addition, the difference between the lowest value obtained during each common rest (R1 and R2) and pre-exercise value were calculated. Only the ΔSBP was analyzed because photoplethysmographic measurement of BP during resistance exercise was validated by comparison with intra-arterial measurements only for the SBP increase during exercise (Δ) and not for the DBP nor the absolute values achieved during exercise (Gomides et al., 2010b).

RPE was assessed every 15 repetitions during the exercise by the Omni scale (Robertson et al., 2003) with 0 meaning “extremely easy” and 10 “extremely hard.” Blood lactate concentration was measured pre- and at 3 and 5 min after the exercises, and was analyzed by electrochemical technique (Lactate Analyzer, Yellow Springs Instruments 2300 Stat Plus, OH, United States).

### Statistical Analyses

Data normality was verified by the Shapiro-Wilk test and visual inspection. Reliability of 1RM for each exercise was checked by the intraclass correlation coefficient (ICC – 95% confidence interval) calculated between the values obtained at the two last assessing sessions. For each exercise, the protocol duration and the areas under the curve for SBP, HR, and RPP were compared between the 3 × 15:88 s and 9 × 5:22 s protocols using paired *t*-tests. In addition, for each exercise, a two-way ANOVA for repeated measurements was used to compare ΔSBP, ΔHR, ΔRPP, RPE, and lactate concentration between the RTPs (3 × 15:88 s and 9 × 5:22 s) and the phases (1–15 rep, R1, 16–30 rep, R2, and 31–45 rep; or pre-exercise, 3 and 5 min). When necessary, the *post hoc* Newman-Keuls test was applied. The effect size for the main results was calculated for ΔSBP, ΔHR, and ΔRPP using Cohen’s d procedure. = 3 × 15:88 s mean – 9 × 5:22 s mean/SD_pooled_, where spooled = √[(SD${}_{3\times 15:88\,\text{s}}^{2}$ + SD${}_{9\times 5:22\,\text{s}}^{2}$)/2] (Cohen, 1988). Thus, we have calculated the effect sizes of these delta values between the different exercise protocols. The effect size was classified as small (0.0 ↔ 1.2), moderate (1.2 ↔ 1.9), or large (>2.0). A *post hoc* power analysis was calculated using the G Power software (version 3.1.4, Heinrich Heine University, Düsseldorf, Germany). For a sample size of 12, and a large effect, statistical power to BLE ranged from 0.59 to 0.83 and to UEF ranged from 0.39 to 0.46 depending on the statistical test. The adopted significance level was *p* < 0.05. Data are presented as mean ± standard deviation (SD).

## Results

Thirty-two participants wanted to participate in the study and signed the consent form. However, during the examinations in the Hypertension Unit, 16 volunteers were identified as not meeting all the study criteria because seven of them had comorbidities associated with hypertension (four diabetes, one heart failure, one thalassemia, and one stroke), one was obese (BMI > 35 kg.m^–2^), two were athletes, one was taking four classes of medication, three were taking beta-blockers and two were diagnosed as normotensive. Additionally, four participants reported lack of interest in participating after doing this preliminary examination. Thus, 12 participants completed all study procedures (Table 1). Most of the sample was composed by women (66.7%) and presented as overweight (BMI > 25 kg. m^–2^). Nine participants were taking only one class of antihypertensive medication, while the other three took two classes.

**TABLE 1:** Participants’ characteristics.

Characteristic	*N* = 12
Sex (male/female)	4/8
Age (years)	48.4 ± 7.8
Height (cm)	162 ± 9
Weight (kg)	77.8 ± 15.3
Body mass index (kg/m^2^)	29.5 ± 3.7
Rest SBP with medication (mmHg)	132 ± 13
Rest DBP with medication (mmHg)	84 ± 8
**Anti-hypertensive medication**	
Alpha-1 adrenergic blocker	6 (50%)
Angiotensin-converting enzyme inhibitor	2 (17%)
Calcium channel antagonist	1 (8%)
Angiotensin-converting enzyme inhibitor + Diuretic	2 (17%)
Calcium channel antagonist + Angiotensin-converting	1 (8%)
enzyme inhibitor	

The reliability of 1RM assessment was high for both exercises (BLE – ICC = 0.999, 0.998–1.000, *p* < 0.000 and UEF – ICC = 0.998, 0.994–0.999, *p* < 0.000). The mean 1RM value was 41 ± 7 kg for BLE and 11 ± 4 kg for UEF.

### Responses to Bilateral Leg Extension (BLE) Exercise

**FIGURE 2:**
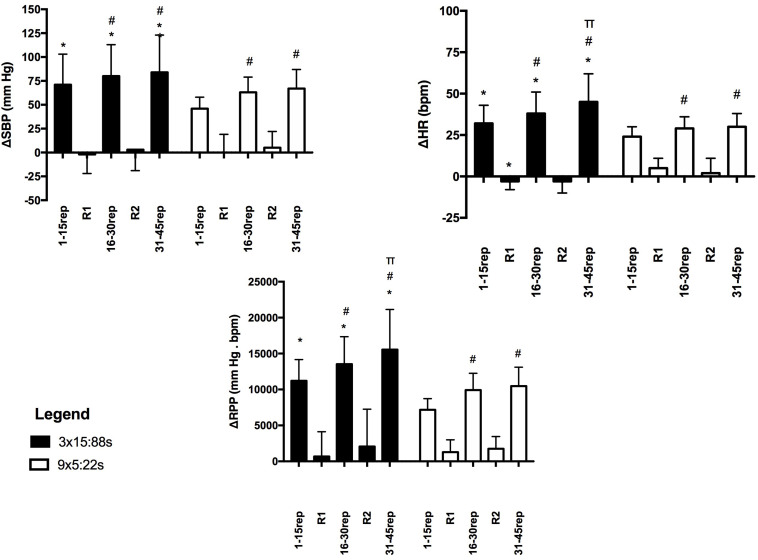
Greatest changes in systolic blood pressure (ΔSBP), heart rate (ΔHR), and rate pressure product (ΔRPP) measured during the bilateral leg extension exercise executed with the 3 × 15:88 s and the 9 × 5:22 s protocols in hypertensive participants. 1–15 rep = 1st and 15th repetitions; 16–30 rep = 16th and 30th repetitions; 31–45 rep = 31st and 45th repetitions; R1 = first common rest interval; R2 = second common rest interval. *Different from 9 × 5:22 s at the same phase (*p* < 0.05). ^#^Different from 1 to 15 rep at the same protocol (*p* < 0.05). ^π^Different from 16 to 30 rep at the same protocol (*p* < 0.05).

Concerning the peak responses for BLE exercise (Figure 2) significant interactions between RTP and phase were found for ΔSBP (*F* = 8.60, *p* < 0.001, power = 0.807), ΔHR (*F* = 16.28, *p* < 0.001, power = 0.873), and ΔRPP (*F* = 12.10, *p* < 0.001, power = 0.871). Thus, ΔSBP, ΔHR and ΔRPP were significantly greater in the 3 × 15:88 s than in the 9 × 5:88 s for all blocks of repetitions (1–15 rep, 16–30 rep, and 30–45 rep). In addition, ΔSBP was significantly higher in the second and third blocks than in the first block of repetitions, while ΔHR and ΔRPP increased progressively across the blocks only in the 3 × 15:88 s. For the rest intervals, ΔSBP, ΔHR, and ΔRPP were similar between the protocols and between the common rests (R1 and R2), except for ΔHR in the first common rest (R1) that was lower in the 3 × 15:88 s protocol.

The effect sizes of ΔSBP, ΔHR, and ΔRPP to BLE exercise are shown in Table 2. For ΔSBP, the effect sizes were small for all phases. For ΔHR the effect sizes were moderate for R1 and small for other phases. For ΔRPP the effect sizes were moderate for 1–15 rep and small for other phases.

**TABLE 2:** Effect sizes (Cohen’s d) and their confidence interval (95% CI) for comparison of the increase in systolic blood pressure (ΔSBP), heart rate (ΔHR), and rate pressure product (ΔRPP) between the two resistance training protocols 3 × 15:88 s and 9 × 5:22 s in the bilateral leg extension and unilateral elbow flexion exercise.

	Bilateral leg extension exercise						Unilateral elbow flexion exercise					
	ΔSBP		ΔHR		ΔRRP		ΔSBP		ΔHR		ΔRRP	
	Cohen’s d	95% CI (p)	Cohen’s d	95% IC (p)	Cohen’s d	95% IC (p)	Cohen’s d	95% IC (p)	Cohen’s d	95% IC (p)	Cohen’s d	95% IC (p)
1–15 rep	1.0	0.1**↔**1.8 (*p* = 0.00)	0.9	0.0**↔**1.7 (*p* = 0.03)	1.7	0.7**↔**2.6 (*p* = 0.02)	0.5	−0.4**↔**1.2 (*p* = 0.43)	0.3	−0.5**↔**1.1 (*p* = 0.18)	0.4	−0.4**↔**1.2 (*p* = 0.43)
R1	–0.1	−0.9**↔**0.7 (*p* = 0.43)	–1.4	−2.3**↔**−0.5 (*p* = 0.28)	–0.2	−1.0**↔** 0.6 (*p* = 0.01)	–0.2	−0.9**↔**0.6 (*p* = 0.21)	–1.3	−2.1**↔**−0.4 (*p* = 0.01)	–1.0	−1.7**↔**0.1 (*p* = 0.22)
16–30 rep	0.7	−0.2**↔**1.4 (*p* = 0.01)	0.9	0.0**↔**1.7 (*p* = 0.03)	1.1	0.2**↔**2.0 (*p* = 0.06)	0.2	−0.6**↔**1.0 (*p* = 0.43)	0.1	−0.7**↔** 0.9 (*p* = 0.03)	0.1	−0.7**↔**0.9 (*p* = 0.25)
R2	–0.1	−0.9**↔**0.7 (*p* = 0.20)	–0.6	−1.4**↔**0.2 (*p* = 0.21)	0.1	−0.7**↔**0.9 (*p* = 0.00)	–0.3	−1.1**↔**0.5 (*p* = 0.14)	–0.5	−1.3**↔** 0.4 (*p* = 0.18)	–0.7	−1.5**↔** 0.1 (*p* = 0.47)
31–45 rep	0.5	−0.3**↔**1.3 (*p* = 0.02)	1.1	0.2**↔**1.9 (*p* = 0.01)	1.2	0.3**↔**2.0 (*p* = 0.01)	0.4	−0.4**↔**1.2 (*p* = 0.19)	0.1	−0.7**↔**0.9 (*p* = 0.10)	0.3	−0.5**↔**1.1 (*p* = 0.20)

The area under the curve for SBP, HR, and RPP did not differ between the protocols 3 × 15:88 s and 9 × 5:22 s (56,396 ± 7,981 vs. 55,359 ± 6,731 mm Hg. s; 31,845 ± 4,920 vs. 31,023 ± 3,690 bpm. s; and 5,260 ± 937 vs. 5,082 ± 683 mm Hg. bpm. s. 10^3^, respectively, all *p* > 0.310).

Concerning RPE, during BLE, regardless of the phase (RTP main effect: *F* = 5.96, *p* < 0.05, power = 0.605), it was significantly higher in the 3 × 15:88 s than in the 9 × 5:22 s protocol. The combined values were 6.7 ± 2.8 vs. 5.8 ± 3.0, respectively. In addition, regardless of the protocol (phase main effect: *F* = 13.18; *p* < 0.00, power = 0.827), RPE increased significantly at the last two repetition blocks in comparison to the first block (16–30 rep and 31–45 rep > 1–15 rep) (Table 3). The combined values were 1–15 rep = 5.5 ± 2.7, 16–30 rep = 6.4 ± 3.0 and 31–45 rep = 7.2 ± 2.9. Blood lactate concentrations increased similarly in the post-exercise period (3 and 5 min) in comparison to pre-exercise in both RTPs (phase main effect: *F* = 8.49, *p* < 0.001, power = 0.628) (Table 3). The combined values were pre-exercise = 0.88 ± 0.33, 3 min post-exercise = 1.65 ± 1.10 and 5 min post-exercise = 1.61 ± 0.95 mmol. l^–1^).

**TABLE 3:** Rate of perceived exertion (RPE) and lactate concentration measured during the execution of the bilateral leg extension and the unilateral elbow flexion exercises executed with the two resistance training protocols (3 × 15:88 s and the 9 × 5:22 s) in hypertensive participants.

	Bilateral leg extension exercise		Unilateral elbow flexion exercise	
	3 × 15:88 s	9 × 5:22 s	3 × 15:88 s	9 × 5:22 s
**RPE (0–10)**				
1–15 rep	6.0 ± 2.5(*)	4.8 ± 2.9	4.6 ± 2.9	4.4 ± 2.6
16–30 rep	6.8 ± 2.9(*^#^)	5.9 ± 3.0(^#^)	5.4 ± 3.1(^#^)	5.1 ± 2.7(^#^)
31–45 rep	7.3 ± 2.9(*^#^)	6.6 ± 2.9(^#^)	6.0 ± 3.2(^#π^)	5.8 ± 2.8(^#π^)
**Lactate (mmol. l^–11^)**				
Pre-Lac	0.9 ± 0.3	0.9 ± 0.3	0.8 ± 0.2	0.9 ± 0.3
Post-Lac 3 min	1.8 ± 1.2^(&)^	1.5 ± 0.9^(&)^	1.0 ± 0.5	1.0 ± 0.3
Post- Lac 5 min	1.7 ± 1.2^(&)^	1.5 ± 0.7^(&)^	1.2 ± 0.5*^&^	0.8 ± 0.3

Rep = repetitions, Pre-Lac = pre-exercise lactate; post-lac = post-exercise lactate, 3 × 15:88 s and 9 × 5:22 s = sets x reps: rest between sets. *Different from 9 × 5:22 s at the same phase (p < 0.05). ^#^Different from 1 to 15 rep at the same RTP (p < 0.05). ^π^Different from 16 to 30 rep at the same RTP (p < 0.05). ^&^Different of Pre-Lac. () main effect in ANOVA.

### Responses to Unilateral Elbow Flexion (UEF) Exercise

**FIGURE 3:**
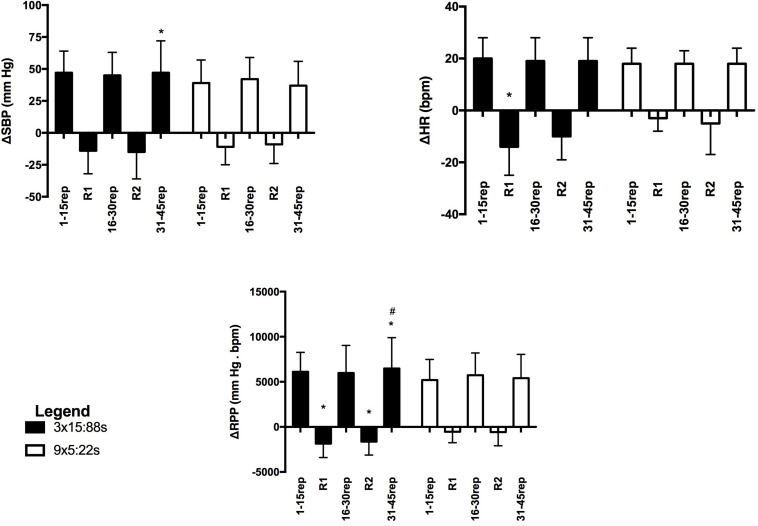
Greatest changes in systolic blood pressure (ΔSBP), heart rate (ΔHR), and rate pressure product (ΔRPP) measured during the unilateral elbow flexion exercise executed with the 3 × 15:88 s and the 9 × 5:22 s protocols in hypertensive participants. 1–15 rep = 1st and 15th repetitions; 16–30 rep = 16th and 30th repetitions; 31–45 rep = 31st and 45th repetitions; R1 = first common rest interval; R2 = second common rest interval. *Different from 9 × 5:22 s at the same phase (*p* < 0.05). ^#^Different from 1 to 15 rep at the same protocol (*p* < 0.05). ^π^Different from 16 to 30 rep at the same protocol (*p* < 0.05).

For the UEF exercise (Figure 3), significant interactions between RTP and phase were found for ΔSBP (*F* = 3.82, *p* < 0.001, power = 0.575), ΔHR (*F* = 3.98, *p* < 0.001, power = 0.650), and ΔRPP (*F* = 6.08, *p* < 0.001, power = 0.814). ΔSBP and ΔRPP were significantly greater in the 3 × 15:88 s than in the 9 × 5:22 s protocol in the last block of repetitions (31–45 rep). ΔHR was significantly lower in the 3 × 15:88 s than in the 9 × 5:22 s in R1. ΔRPP was significantly lower in the 3 × 15:88 s than in the 9 × 5:22 s in R1 and R2. In addition, in the 3 × 15:88 s protocol, ΔRPP was higher in the third than in the first block of repetitions.

The effect sizes of ΔSBP, ΔHR, and ΔRPP to UEF exercise are shown in Table 2. For ΔSBP and ΔRPP, the effect size was small for all phases. Whereas, ΔHR presented moderate effect size for R1 and small for other phases.

The area under the curve for SBP, HR, and RPP did not differ between the protocols 3 × 15:88 s and 9 × 5:22 s (51,539 ± 6,411 vs. 51,552 ± 8,180 mm Hg. s; 29,605 ± 3,842 vs. 30,233 ± 3,698 bpm. s; and 4,338 ± 358 vs. 4377 ± 586 mm Hg. bpm. s. 10^3^, respectively, all *p* > 0.255).

Concerning RPE, during UEF, only a main effect for phase was observed (*F* = 21.0, *p* < 0.001, power = 0.988). Thus, regardless of the protocol, RPE increased progressively throughout the blocks of 15 repetitions (31–45 rep > 16–30 rep > 1–15 rep) (Table 3). The combined values were 1–15 rep = 4.5 ± 2.7, 16–30 rep = 5.2 ± 2.9 and 31–45 rep = 5.9 ± 3.0. For lactate concentration, there was a significant interaction between RTP and phase (*F* = 5.70; *p* < 0.01; power = 0.601). Lactate concentration increased significantly at 5 min after the 3 × 15:88 s protocol in comparison to pre-exercise, and this increase was significantly greater than 9 × 5:22 s (Table 3).

## Discussion

The aim of this study was to compare the acute cardiovascular responses of medicated hypertensive individuals to two work to rest equated-RTP in exercises involving upper or lower limbs. The main findings were that for both, BLE and UEF, the 3 × 15:88 s RTP produced a greater increase in SBP than the 9 × 5:22 s. In addition, for BLE, the 3 × 15:88 s also produced a greater increase in HR and RPP.

A large and sharp increase in SBP is observed during the execution of a resistance exercise and may trigger the rupture of a pre-existent aneurism, which is especially important to hypertensives who are more prone to developing such an event (Lewington et al., 2002). However, few studies have investigated the cardiovascular responses to RTP in hypertensives (Nery et al., 2010; Souza et al., 2015). One of them have shown that non-medicated hypertensives presented greater increases in SBP during a RTP than normotensives (Nery et al., 2010). In addition, BP increase during RTP is blunted when hypertensives were medicated (Souza et al., 2015), suggesting that medication protects these individuals during RTP. The present study expands this previous knowledge by showing that changing the RTP structure can also protect hypertensive individuals.

By changing the protocol organization (more sets with less repetitions) without changing its work to rest ratio, the present study showed a way to blunt SBP peak increase during RTP. Interestingly, despite the fact that the 3 × 15:88 s protocol has an organization of sets and rest intervals compatible with hypertension guidelines (Williams et al., 2007; Thompson et al., 2013; Malachias et al., 2016), it produced a peak SBP increase approximately 20 mm Hg greater than the 9 × 5:22 s protocol during BLE and 10 mm Hg greater during UEF. It is important to note that although peak ΔSBP was greater in the 3 × 15:88 s protocol, the areas under the curve of SBP were similar between the protocols. The area under the curve represents the total cardiovascular overload imposed by the protocol throughout its execution, while the peak response represents the acute peak load imposed to the cardiovascular system. Thus, although both protocols produced the same total overload, the 3 × 15:88 s protocol resulted in a greater abrupt increase in SBP (Vlak et al., 2012). Therefore, the results of the present study may have an important clinical implication suggesting that the protocol 9 × 5:22 s is safer for hypertensives than the protocol usually suggested in the hypertension guidelines.

The change in RTP organization kept the same volume, load, and duration of the exercises, but decreased ΔSBP during both upper and lower limb exercises. However, the effect was more evident during the lower limb exercise, since in BLE the difference between the protocols was observed in all the repetition blocks, while in UEF, it was observed only in the third block. In addition, ΔHR and ΔRPP were greater in 3 × 15:88 s only in the lower limb exercise. The smaller effect of changing the RTP in the upper limb exercise may be related to the lower cardiovascular impact produced by this exercise performed with a smaller muscle mass (Lewis et al., 1985).

The mechanisms behind SBP increase and reduction during, respectively, RTP sets and intervals are beyond the scope of this study, but they have been stated by others and may help explain the results obtained in the present study. When resistance exercise is about to start, central command is activated and promotes vagal inhibition and sympathetic activation (Mitchell et al., 1980). These autonomic adjustments increase cardiac contractility and HR, increasing cardiac output (Lewis et al., 1985). Thereafter, other mechanisms are summed to the central command. The muscle tension in the active region compresses the blood vessels, increasing the vascular resistance in the active muscles (Seals, 1993; Sarelius and Pohl, 2010). In addition, the mechanoreceptors are activated and contribute to the autonomic adjustment. Finally, as exercise goes on, the metaboreflex is activated by the accumulation of metabolites, and produces a further increase in sympathetic activity, promoting vasoconstriction of the inactive regions (Carrington et al., 2003). All these adjustments lead to the large and progressive increase of BP during the resistance exercise execution. During the rest intervals, central command and mechanoreflex are immediately deactivated, while metaboreflex decreases progressively (Carrington et al., 2003; Fisher et al., 2013). In addition, the cessation of muscle contraction produces a large vasodilation in the active muscles (Baum et al., 2003). Consequently, there is a decrease in BP during the rest interval.

Based on these adjustments, it is possible to speculate why the 9 × 5:22 s protocol produced a lower ΔSBP than 3 × 15:88 s, and which mechanisms might be different in BLE and UEF (Toner et al., 1990). It is known that central command activation is mainly related to exercise intensity and to the degree of fatigue (MacDougall et al., 1992), while metaboreflex depends on metabolite accumulation (Fisher et al., 2013). Thus, during BLE, although the exercise intensity was equal between the protocols (50% 1RM), the 3 × 15:88 s protocol had longer sets leading the participants close to fatigue, especially during the second and third blocks of repetitions. This greater fatigue can be seen by the greater RPE in the 3 × 15:88 s protocol, showing that this protocol may have produced a greater activation of the central command (Río-Rodríguez et al., 2016). Interestingly, despite the longer sets, lactate concentration did not differ between the protocols during the BLE, which may be explained by the fact that this protocol has longer rest intervals that may have led to a greater removal of the lactate. Considering UEF, the smaller muscle mass involved in the exercise may have produced a lower stimulus of the central command, as supported by the lower RPE. On the other hand, lactate concentration was greater in the recovery of the 3 × 15:88 s protocol, suggesting a greater activation of the metaboreflex during UEF, which is compatible with the fact that BP difference between the protocols was only observed at the third block of repetitions in which this reflex may be more activated by the accumulation of metabolites.

Some previous studies have reported results in the same direction as the present ones. Although different from the dynamic exercise explored in the present study, Río-Rodríguez et al. (2016) reported that intra-set rest configuration induced lower central and peripheral fatigue during an isometric knee extension exercise, reducing the cardiovascular stress. Additionally, another study comparing W:R equated RTPs (40 × 1:18.5 s vs. 5 × 8:180 s) in healthy participants found lower SBP peaks with the protocol with longer rest intervals (Mayo et al., 2017). Similarly, a previous study from our group involving normotensive participants also reported lower SBP peaks with a 9 × 5:22 s protocol in comparison with a 3 × 15:88 s protocol (Paulo et al., 2019). Together, these results suggest that among RTP equated for W:R, those with shorter sets induce lower cardiovascular stress during execution. However, in our previous study (Paulo et al., 2019), peak SBP was lower in 9 × 5:22 s in comparison to 45 × 1:4 s, showing that set length is not the only factor influencing cardiovascular responses during execution and suggests the need for a balance between sets and interval durations.

### Study Limitations

BP was obtained by an indirect photoplethysmographic method that has been validated against intra-arterial BP measurement only for ΔSBP during RTP (Gomides et al., 2010b). Because of that, the absolute values of SBP/DBP responses were not analyzed. As a first study with hypertensives and to increase the study’s external validity, the participants of the present study were taking different kinds of anti-hypertensive medication as recommended by their own physicians. Thus, the interval between medication use and assessments was not fixed. However, all patients took their medications in the morning before the sessions that were also conducted in the morning. Thus, results may be applied when training was conducted at this time of day. Future studies, however, should compare responses in patients taking different anti-hypertensives and among exercises conducted after different intervals from medication use. We decided to study middle aged participants (30–60 years) because hypertension incidence and prevalence increases at this phase of life and during this period changes associated with the elderly were not present yet. Thus, this is an adequate age group for studying hypertension without the additional concerns derived for aging. Future studies, however, should compare responses in elderly patients. Based on the way RTP is recommended in the guidelines for individuals with cardiovascular disease (Williams et al., 2007), exercise intensity was set as 50% 1RM which may represent a different percentage of the maximal number of repetitions for different individuals (Shimano et al., 2006). Thus, future studies might compare these RTP using a % of the maximum number of repetitions with hypertension.

## Conclusion

In conclusion, in medicated hypertensives, a RTP with 9 × 5:22 s produced a smaller increase in cardiovascular peaks (ΔSBP, ΔHR, and ΔRPP) and the same area under the curve for SBP, HR, and RPP during its execution than a protocol with 3 × 15:88 s.

## Data Availability Statement

The datasets generated for this study are available on request to the corresponding author.

## Ethics Statement

The studies involving human participants were reviewed and approved by Institutional Research Ethics Committee of the Faculty of Medicine of the University of São Paulo (number 110554). The patients/participants provided their written informed consent to participate in this study.

## Author Contributions

AP, VT, and CF contributed to the conception, design, and drafting of the work. AP wrote the first draft of the manuscript. AP, VT, CF, DM, GS, and SB worked on acquisition and interpretation of data. AP, VT, CF, and DM critically reviewed the manuscript for important intellectual content. All authors contributed to the manuscript revision and read and approved the submitted version.

## Conflict of Interest

The authors declare that the research was conducted in the absence of any commercial or financial relationships that could be construed as a potential conflict of interest.
